# Thromboplasminflammation in COVID-19 Coagulopathy: Three Viewpoints for Diagnostic and Therapeutic Strategies

**DOI:** 10.3389/fimmu.2021.649122

**Published:** 2021-06-11

**Authors:** Satoshi Gando, Takeshi Wada

**Affiliations:** ^1^ Acute and Critical Center, Department of Acute and Critical Care Medicine, Sapporo Higashi Tokushukai Hospital, Sapporo, Japan; ^2^ Division of Acute and Critical Care Medicine, Department of Anesthesiology and Critical Care Medicine, Hokkaido University Faculty of Medicine, Sapporo, Japan

**Keywords:** COVID-19, inflammation, plasmin, SARS-CoV-2, thrombin

## Abstract

Thromboplasminflammation in coronavirus disease 2019 (COVID-19) coagulopathy consists of angiotensin II (Ang II)-induced coagulopathy, activated factor XII (FXIIa)- and kallikrein, kinin system-enhanced fibrinolysis, and disseminated intravascular coagulation (DIC). All three conditions induce systemic inflammation *via* each pathomechanism-developed production of inflammatory cytokines. Severe acute respiratory syndrome coronavirus 2 (SARS-CoV-2) downregulates angiotensin-converting enzyme 2, leading to an increase in Ang II levels. Ang II-induced coagulopathy comprising platelet activation, thrombin generation, plasminogen activator inhibitor-1 expression and endothelial injury causes thrombosis *via* the angiotensin II type 1 receptor. SARS-CoV-2 RNA and neutrophil extracellular trap (NET) DNA activate FXII, resulting in plasmin generation through FXIIa- and kallikrein-mediated plasminogen conversion to plasmin and bradykinin-induced tissue-type plasminogen activator release from the endothelium *via* the kinin B2 receptor. NETs induce immunothrombosis at the site of infection (lungs), through histone- and DNA-mediated thrombin generation, insufficient anticoagulation control, and inhibition of fibrinolysis. However, if the infection is sufficiently severe, immunothrombosis disseminates into the systemic circulation, and DIC, which is associated with the endothelial injury, occurs. Inflammation, and serine protease networks of coagulation and fibrinolysis, militate each other through complement pathways, which exacerbates three pathologies of COVID-19 coagulopathy. COVID-19 coagulopathy causes microvascular thrombosis and bleeding, resulting in multiple organ dysfunction and death in critically ill patients. Treatment targets for improving the prognosis of COVID-19 coagulopathy include thrombin, plasmin, and inflammation, and SARS-CoV-2 infection. Several drugs are candidates for controlling these conditions; however, further advances are required to establish robust treatments based on a clear understanding of molecular mechanisms of COVID-19 coagulopathy.

## Introduction

Based on the knowledge of severe acute respiratory syndrome (SARS), analyses of full-length genome sequences of novel coronavirus revealed that they share 79.6% and 96% sequence identity with SARS coronavirus (SARS-CoV) and to bat coronavirus, respectively (1). These studies confirmed that novel coronaviruses use the same angiotensin-converting enzyme 2 (ACE2) as SARS-CoV as entry receptor to the infected cells (1, 2),. Accordingly, the novel coronavirus is named SARS-CoV-2 and the disease caused by SARS-CoV-2 is called coronavirus disease 2019 (COVID-19).

A rampage through the body (3). SARS-CoV-2 affects multiple organs in the body, from the brain to the blood, and is not limited to the lungs (3). Above all, the blood is one of the organs that has attracted much attention worldwide. Reports from China showed higher deterioration in platelet counts, coagulation and fibrinolysis markers, especially in D-dimer levels in non-survivors than in survivors of COVID-19 (4, 5). Tang et al. (6) showed a high incidence (71,4%) of disseminated intravascular coagulation (DIC) and abnormally high levels of fibrin/fibrinogen degradation products (FDP) and D-dimer in non-survivors of COVID-19. Following these studies, the International Society on Thrombosis and Haemostasis published interim guidance on COVID-19 coagulopathy (7). Since this announcement, an exceptionally high incidence of thromboembolic complications associated with abnormal coagulation parameters in COVID-19 has been spread (8). Importantly, bleeding complications were also high, and the odds ratio of D-dimer for prediction of bleeding was 3.56 in critically ill patients with SARS-CoV-2 infection (9). Contrary to the findings of Tang et al. (6), DIC was rare (2.2%) but was associated with significant bleeding in this study (9). The results coincide with the notion that DIC has long been acknowledged as a “thrombohemorrhagic disorder” (10). However, laboratory findings of early phase of COVID-19 show a moderate decrease in platelet counts, mild prolongation of prothrombin time, and high fibrinogen levels, which are different from DIC, and leads us to consider other pathomechanisms of COVID-19 coagulopathy (11).

The three key words of COVID-19 coagulopathy are inflammation (12), thrombosis, and bleeding. We tried to unify these key words explaining three viewpoints; ACE2 participated in angiotensin II (Ang II)-induced coagulopathy, hyperfibrinolysis, and DIC. Thromboplasminflammation in COVID-19 coagulopathy for diagnostic and therapeutic implications will be discussed based on these three viewpoints in this review.

## Methods

PubMed was searched using terms (COVID-19 or SARS-CoV-2) and (ACE2 or angiotensin II, inflammation, coagulopathy, coagulation, fibrinolysis, thrombin, plasmin, treatment, guidance or guidelines) for studies published from January 1, 2020, to May 20, 2021. We manually checked the references of the selected articles to identify additional relevant articles. Articles relevant to the three keywords and viewpoints were selected and discussed.

## Underlying Findings

### Coronavirus Family and Coagulopathy

In addition to SARS-CoV and SARS-CoV-2, Middle East respiratory syndrome coronavirus (MERS-CoV), influenza viruses (including seasonal A and B, avian H5N1, and swine H1N1, etc.), and human coronavirus NL63 belong to the human coronavirus family. MERS-CoV uses human dipeptidyl peptidase-4 as the entry receptor, whereas the other viruses invade cells through ACE2 (13). Main cell types infected by these coronaviruses are airway epithelial cells and lung type II pneumocytes which cause pneumonia and, in severe cases, acute respiratory distress syndrome (ARDS). Importantly, all coronaviruses give rise to variety degree of thrombocytopenia, coagulation activation, and inhibition of fibrinolysis or hyperfibrinolysis, thus increasing the risk of local and systemic thrombosis, microvascular thrombosis, and bleeding (14, 15). Although similarities and differences in mechanisms of coagulofibrinolytic changes in coronaviruses have been discussed, ACE2 and the renin-angiotensin-aldosterone system (RAAS) is considered one of main mechanisms of coagulopathies in SARS-CoV, SARS-CoV-2, influenza virus, and human coronavirus NL63 infections (13–15).

### SARS-CoV-2 Infection and the Renin-Angiotensin-Aldosterone System

**Figure 1:**
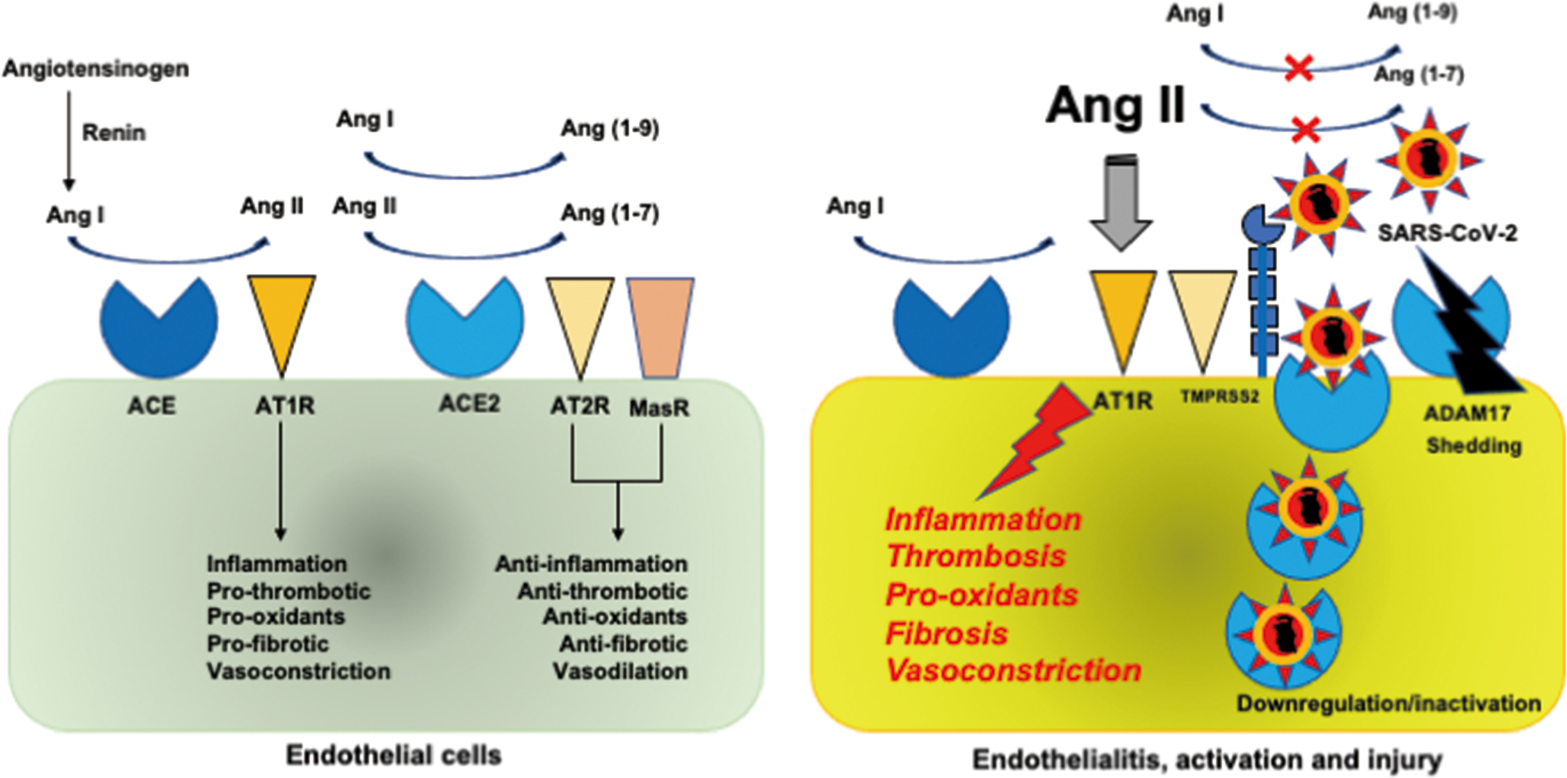
Renin-angiotensin-aldosterone system. (Left). Renin converts angiotensinogen to Ang I, which is converted to Ang II by ACE. ACE2 degrades Ang I and Ang II to generate Ang(1-9) and Ang(1-7), which have anti-inflammatory and antithrombotic actions through AT2R and MasR. Conversely, Ang II has proinflammatory and prothrombotic properties binding to AT1R and secretes aldosterone from the adrenal cortex. RAAS is balanced by its two opposed properties of inflammation and coagulation by ACE2. (Right). Binding of SARS-CoV-2 to ACE2 *via* the spike glycoprotein to TMPRSS2 results in the downregulation and inactivation of ACE2, leading to an increase in Ang II levels, which gives rise to inflammation and thrombosis through AT1R. Proteolysis and shedding of ACE2 resulting from ADAM17 upregulation also contributes to inactivation of ACE2. SARS-CoV-2 infection of endothelial cells causes endothelialitis associated with endothelial activation and injury. ACE, angiotensin-converting enzyme; ADAM17, a disintegrin and metalloprotease domain-containing protein 17; Ang, angiotensin; AT1R, angiotensin II type 1 receptor; MasR, Mas receptor; RAAS, renin-angiotensin-aldosterone system; SARS-CoV-2, severe acute respiratory syndrome coronavirus 2; TMPRSS2, transmembrane protease serine type 2.

The SARS-CoV-2 is a single-stranded RNA virus with an envelope that uses ACE2 as a cell entry receptor through envelope-anchored spike glycoprotein binding (1, 16). Spike glycoprotein drives the fusion of viral and infected cell membranes using S2 subunit through cleavage at the S1/S2 and S2’ sites of glycoprotein by furin and transmembrane protease serine type-2 (TMPRSS2), respectively (16, 17). The RAAS, which includes ACE2, has long been well known (**Figure 1**). Renin converts angiotensinogen to angiotensin I (Ang I), which is converted to Ang II by ACE. ACE2 degrades Ang I and Ang II to generate Ang(1-9) and Ang(1-7), which have anti-inflammatory and antithrombotic actions through the angiotensin II type 2 receptor (AT2R) and Mas receptor (18, 19). Conversely, Ang II has proinflammatory and prothrombotic properties binding to AT1R and secretes aldosterone from the adrenal cortex. Accordingly, RAAS is balanced by its two opposing properties of inflammation and coagulation by ACE2.

ACE2 is expressed in various cells of the whole body, including the lung epithelial cells, and endothelial cells (20). Binding of SARS-CoV-2 to ACE2 results in the downregulation and inactivation of ACE2, leading to an increase in Ang II levels (18). Liu et al. (21) confirmed the elevation of Ang II levels in parallel with the SARS-CoV-2 viral load in patients with COVID-19. Increased Ang II causes inflammation, thrombosis, and other negative effects on the body. Downregulation and inactivation of ACE2 occur mainly because of endocytosis through binding of the SARS-CoV-2 spike glycoprotein to ACE2 (1, 2, 16, 17). Proteolysis and shedding of ACE2 resulting from the upregulation of a disintegrin and metalloprotease domain-containing protein 17 (ADAM17) also contributes to ACE2 inactivation (22). The TMPRSS2-induced cleavage of ACE2 observed in SARS-CoV may play a role in shedding of ACE2 during SARS-CoV-2 infection (23). It has been speculated that there is an interplay between SARS-CoV-2 and interferons. Studies on SARS-CoV and human coronavirus NL-63 suggest that SARS-CoV-2 may induce interferon production, which downregulates ACE2 (24, 25). However, one study showed that interferon induces ACE2 expression (26). It was subsequently demonstrated that a truncated ACE2 isoform, not the full-length active ACE2, was induced by interferon (27). Therefore, the role of interferon in ACE2 downregulation in SARS-CoV-2 infection remains to be elucidated. Viral replication-dependent ACE2 downregulation during the culture of human coronavirus NL63 at optimal temperature suggests that viral load may have a role in the lack of availability of ACE2 in COVID-19 (28).

### Pyroptosis and Necroptosis

Pathogen-associated molecular patterns (PAMPs) and damage-associated molecular patterns (DAMPs) are the ligands of innate immune system pattern recognition receptors such as toll-like receptors (TLRs), nucleotide-binding oligomerization domain-containing (NOD)-like receptors (NLRs), and retinoic acid-inducible gene I (RIG-I)-like receptors (RLRs). PAMPs, such as bacterial lipopolysaccharide or viral RNA, induce the lytic programmed cell death processes called pyroptosis and necroptosis resulting in cell death and releases of DAMPs such as nucleic acids (DNA/RNA), histones, mitochondrial DNA, and high mobility group box 1 (HMGB1), all of which are involved in changes in inflammation, coagulation, and fibrinolysis (29, 30). Therefore, there is bidirectional interplay between PAMPs and DAMPs *via* pyroptosis and necroptosis.

Pyroptosis is caused by procaspase-1 and/or procaspase-11 (human capase-4 and -5) combining with NOD-like receptor family-involved pyrin domain-containing 3 (NLRP3) to generate the NLRP3 inflammasome, which then cleaves gasdermin D (GSDMD), releasing its N- and C-terminal products (GSDMD-N and GSDMD-C) through either direct activation of caspase-1 or indirectly through the recruitment of adaptor protein, apoptosis-associated speck-like protein containing a caspase activating and recruitment domain (ASC). GASDMD-N disrupt cell membrane by pore formation (29, 30).

The necroptosis pathway is induced by toll/interleukin-1 (IL-1) receptor homology domain-containing adaptor inducing interferon-β (TRIFF)-dependent activation of TLR3, and TLR4, and interferon receptor signaling. Autophosphorylation of receptor interacting serine/threonine kinase 1 (RIPK1) recruits RIPK3 resulting in mixed lineage kinase domain-like (MLKL) activation, leading to translocation of MLKL to plasma membranes followed by membrane disruption by permeabilization (29–31). Apoptotic caspases-3 and -8 mediate interplays among apoptosis, pyroptosis, and necroptosis (31). Although necroptosis occurs after impairment of apoptosis as backup mechanism, pyroptosis is considered as primary responses to cellular insult (30, 31).

Cell death programs are primarily physiological innate immune inflammatory responses against insults including infection through the protection from pathogen spreading by inducing death of infected cells, and through production of DAMPs, which evoke further inflammation and coagulation, in addition to PAMPs. However, when the insults are extensively severe, over-activated pyroptosis and necroptosis give rise to pathological phenomena (29, 32, 33). SARS-CoV-2 binding to ACE2, and Ang II-induced activation of AT1R, and activation of complement pathways by SARS-CoV-2 may induce assembly of the NLRP3 inflammasome (33–35), which contributes to the onset of cytokine storm, pneumonia, ARDS, thrombosis, DIC, and other types of organ dysfunction (35–37).

### Cytokines

Viral RNA, function as PAMPs, and infected cell pyroptosis- and necroptosis-generated DAMPs bind to pattern recognition receptors such as TLR3, TLR7/TLR8, RLRs, and NLRP3, which induce the transcription of proinflammatory cytokines and type-1 interferon-α and -β (38, 39). High plasma levels of tumor necrosis factor-α (TNF-α), IL-1β, IL-6, and IL-8 have been confirmed in COVID-19, especially in non-survivors and in critically ill patients (4, 12, 40, 41). SARS-CoV-2-activated inflammatory cells, such as macrophages, monocytes, and neutrophils are thought to be the producing cells of these inflammatory cytokines (12, 42).

### Platelets and Neutrophils Activation

DAMPs as well as inflammatory cytokines induce platelet activation and subsequent aggregation directly or through interactions with TLR2 and TLR4 expressed on the platelets, leading to platelet-neutrophil interactions (43–45). In addition, numerous viruses directly activate platelets *via* a variety of platelet surface receptors such as TLR7, FCγRII, and glycoprotein IIb/IIIa (46). Influenza virus, which is a single-stranded RNA virus, same as SARS-CoV-2, activates platelet *via* TLR7 (47). The activated platelets upregulate P-selectin from α-granules and express CD40 ligand (CD40L), leading to consequent their binding to P-selectin glycoprotein ligand-1 and CD40 on monocytes, neutrophils, and endothelial cells (48, 49). These processes release　neutrophil extracellular traps (NETs) from neutrophils and express selectins and other adhesion molecules on endothelial cells.

Higher levels of soluble P-selectin and soluble CD40L in COVID-19 patients admitted to ICU than in non-ICU patients indicate platelet activation in severe COVID-19 (50). Lower platelet counts in non-survivors and severe cases of COVID-19 than in survivors and non-severe cases support platelet activation and its consumption (51, 52). Viruses can induce NET formation, which has roles in the pathogenesis of viral infection-induced systemic pathologies such as vasculitis and DIC (53). High-dimensional flow cytometric marker analysis revealed that neutrophil and platelet activations are associated with platelet-neutrophil aggregates in the blood of COVID-19 pneumonia patients (54). Myeloperoxidase-DNA and citrullinated histone H3, surrogate markers of NET formation, showed high levels in COVID-19, especially in patients on ventilators or admitted to the ICU (55, 56).

Taken together, SARS-CoV-2 induces activation and interactions of platelets and neutrophils, which give rise to NET release, associated with high levels of histones in the circulation.

### Endothelial Cell Activation and Injury

TNF-α and IL-1 upregulate P-selectin and induce the expression of E-selectin on the endothelium and L-selectin on the neutrophils, leading to neutrophil and endothelial interactions, which accelerates the expression of intercellular adhesion molecule-1 (ICAM-1) and vascular cell adhesion molecule-1 (VCAM-1) in the endothelial cells (43, 57, 58). These processes promote activated neutrophils to adhere to endothelial cells secreting elastase, metalloproteases, myeloperoxidase, and reactive oxygen species, then induce endothelial injury (57, 59, 60). NETs containing histone directly injure endothelial cells. Sera from patients with high levels of histones showed reduced viability of cultured endothelial cells (61). Histones bind to endothelial cells and then induce Ca^2+^ influx and overload with consequent pore formation, leading to endothelial injury (62).

High levels of von Willebrand Factor antigen and its activity, and factor VIII (FVIII) activity in ICU-admitted COVID-19 patients indicate endothelial activation (50). Low survival provability in COVID-19 patients with elevated levels of soluble thrombomodulin, a marker of endothelial injury, suggest endothelial injury in severe COVID-19 cases (50). Scanning electron micrographs of pulmonary autopsy specimens revealed endothelial destruction and SARS-CoV-2 invasion in the endothelial cells, which suggest viral endothelialitis-induced endothelial injury (63).

Briefly, SARS-CoV-2, SARS-CoV-2-induced inflammatory cytokine expression, activated neutrophil-released granule-containing elastase, reactive oxygen species, and NETs with histones cause endothelial cell activation and injury.

### Systemic Inflammation, Macro, and Microvascular Thrombosis

Large cohort studies on COVID-19 confirmed high temperature, and increased heart rate, respiratory rate, and white blood cell counts in severe cases and non-survivors, suggesting systemic inflammation with high levels of proinflammatory cytokines (4, 64). These have been recognized as systemic inflammatory response syndrome (SIRS) or cytokine storm, which are associated with systemic cytokine circulation, prothrombotic state, and endothelial dysfunction with increased permeability (10, 42, 65).

In addition to the high incidence of rapid arterial and deep venous thrombosis formation, microvascular fibrin thrombosis is a prominent characteristic of COVID-19 (7–9, 66). The first target of SARS-CoV-2 infection is the lung; therefore, many autopsy studies revealed fibrin microvascular thrombosis in arterioles, venules, and capillaries in the lungs (63, 67, 68). Pulmonary vascular occlusion from capillaries to medium-sized vessels with aggregated NETs were also confirmed (56). This stage of lung injury is called type L ARDS and is characterized by hypoxemia with low elastance (69). Importantly, in accordance with disease progression, thrombosis with neutrophilic plugs were present in the brain, heart, liver, kidneys, and spleen, in addition to the lungs (67), which suggests that initial immunothrombosis is disseminated throughout the body (70). SARS-CoV-2-developed pneumonia progresses to ARDS from exudative, proliferative to fibrotic stages showing hyaline membranes, septum thickening, alveolar and microvascular fibrin thrombosis, diffuse alveolar damage, and organizing fibrosis (68). This stage is called type H ARDS and is associated with the same hypoxemia level as that of type L, but is complicated by high elastance owing to diffuse alveolar damage (69). Type H is a classic typical ARDS, which has been known to usually complicate DIC (71).

Taken together, it is hypothesized that type L ARDS at initial infection stage represents pulmonary intravascular coagulopathy comprising immunothrombosis, which progresses to type H ARDS in severe cases at late stage and is associated with systemic microvascular thrombosis in addition to lung thrombosis (69, 72).

### Bleeding

An important finding of an autopsy study was the coexistence of microvascular thrombosis and extensive hemorrhage in the lungs (68). Bleeding events rates in COVID-19 are unexpectedly high at 4.8%, and all patients diagnosed with DIC are associated with high-grade bleeding events with low fibrinogen levels (9). The results were confirmed in another study involving ICU patients, which showed 8.0% bleeding events and 43.3% thrombotic complications (73). A monocenter retrospective study of ICU-admitted patients showed that 21% were associated with hemorrhagic events (74).

### Vaccine-Induced Immune Thrombotic Thrombocytopenia

Vaccine-induced immune thrombotic thrombocytopenia (VITT) in response to the recombinant adenoviral vector encoding the SARS-CoV-2 spike glycoprotein (ChAdOx1 nCov-19) should be considered an important finding related to COVID-19 coagulopathy (75). Cases of thrombosis and thrombocytopenia after vaccination with a replication-incompetent human adenovirus 26 vector vaccine (Ad26.COV2.S) have also been reported (76). Clinical features and laboratory data on instances of VITT resulting in fatal thromboembolic events, DIC, and hemorrhage associated with thrombocytopenia, low fibrinogen, and high D-dimer levels resemble autoimmune heparin-induced thrombocytopenia (75, 77). Platelet factor 4 (PF4)-heparin antibody positivity suggests that interplay between the vaccine and platelets and/or PF4 is a pathological mechanism of VITT (75). Further studies are needed to clarify pathomechanisms and establish diagnostic and therapeutic strategies for specific to VITT management and different from strategies used to address autoimmune heparin-induced thrombocytopenia (75).

## Three Viewpoints

### Ang II-Induced Coagulopathy

SARS-CoV-2 RNAemia was observed in 15% of COVID-19 patients (40). Another study confirmed extremely high frequency of viral RNAemia (78%) in critically ill COVID-19 patients (78). In addition, SARS-CoV-2 was detected in a variety of specimens, including blood (79). Autopsy findings showed systemic SARS-CoV-2 infected cells in multiple organs, including the lungs, heart, kidneys, gastrointestinal tracts, and endothelial cells (63, 67). Elevated Ang II levels being proportional to SARS-CoV-2 viral load are a result of the downregulation of ACE2 existing in a variety of infected organs. Ang II in the infected organs and in the circulation is likely to cause systemic effects on inflammation, platelet function, coagulation, and fibrinolysis through AT1R in COVID-19 (18–21, 80).

#### Platelets

Human platelets express AT1R, and potentiation by Ang II of adrenaline-induced platelet activation is mediated through this receptor (81, 82). Furthermore, another study demonstrated that platelets express ACE2 and TMPRSS2, and that binding of the SARS-CoV-2 spike glycoprotein to ACE2 on platelets could induce αIIb/β3 activation and P-selectin expression, leading to enhancement of thrombosis (83). Ang II infusion into healthy volunteers confirmed platelet activation by elevating plasma β-thromboglobulin levels and platelet surface expression of P-selectin (84). The high levels of Ang II, soluble P-selectin, and soluble CD40L in the circulation suggest the Ang II-induced platelet activation in COVID-19 (21, 50). Confirmation of soluble CD40L in COVID-19 patients with hyperactivated platelet indicate that activation of platelet contribute to activation of CD40-bearing neutrophils (85). Platelet activation, therefore, lead to interactions with neutrophils, resulting in NET formation (70).

#### Coagulation and Fibrinolysis

Ang II induces tissue factor mRNA and antigen expression on monocytes through AT1R (86). Ang II-mediated tissue factor expression in endothelial cells and vascular smooth muscle cells has also been confirmed (87, 88). Ang II accelerates arterial and venous thrombosis development *via* AT1R, which is associated with increased plasma levels of plasminogen activator inhibitor-1 (PAI-1) (89, 90). Furthermore, Ang II infusion to healthy volunteers confirmed the elevation of soluble P-selectin, thrombin and antithrombin complex and prothrombin fragment F1+2 in the plasma (84). These findings suggest that Ang II-induced tissue factor expression leads to systemic thrombin generation, resulting in thrombus formation in the vessels. The promotion of antithrombotic activity by ACE2 activation indirectly supports the idea that downregulation of ACE2 by SARS-CoV-2 participates the Ang II-induced tissue factor-dependent thrombin generation and thrombus formation (91). *In vitro* studies have confirmed that Ang II induced increases in PAI-1 mRNA expression and antigen levels in cultured media of endothelial cells with little changes in tissue-type plasminogen activator (t-PA) levels (87, 92), and these effects were mediated by AT1R (93). Ang II infusion into healthy volunteers result in rapid increases in PAI-1 antigen levels in the circulation (94).

Collectively, it is understood that activation of the tissue factor-dependent coagulation pathway and inhibition of fibrinolysis by Ang II participate in thrombus formation in COVID-19.

#### Endothelial Cells

In addition to PAI-1 production, Ang II has another action on endothelial cells expressing both AT1R and AT2R.　Ang II upregulated the expression of ICAM-1 and VCAM-1 in cultured endothelial cells *via* AT1R (95, 96). Ang II also stimulated the secretion and rapid release of soluble ICAM-1 from endothelial cells both in cultured media and in circulation (96). Endothelialitis with endothelial injury due to SARS-CoV-2 infection may reduce thrombomodulin expression, which causes fibrin deposition as is observed in herpes infection (63, 97). These results suggest that Ang II-induced endothelial cell activation and injury are involved in the mechanisms of thrombus formation in SARS-CoV2 infection.

**Figure 2:**
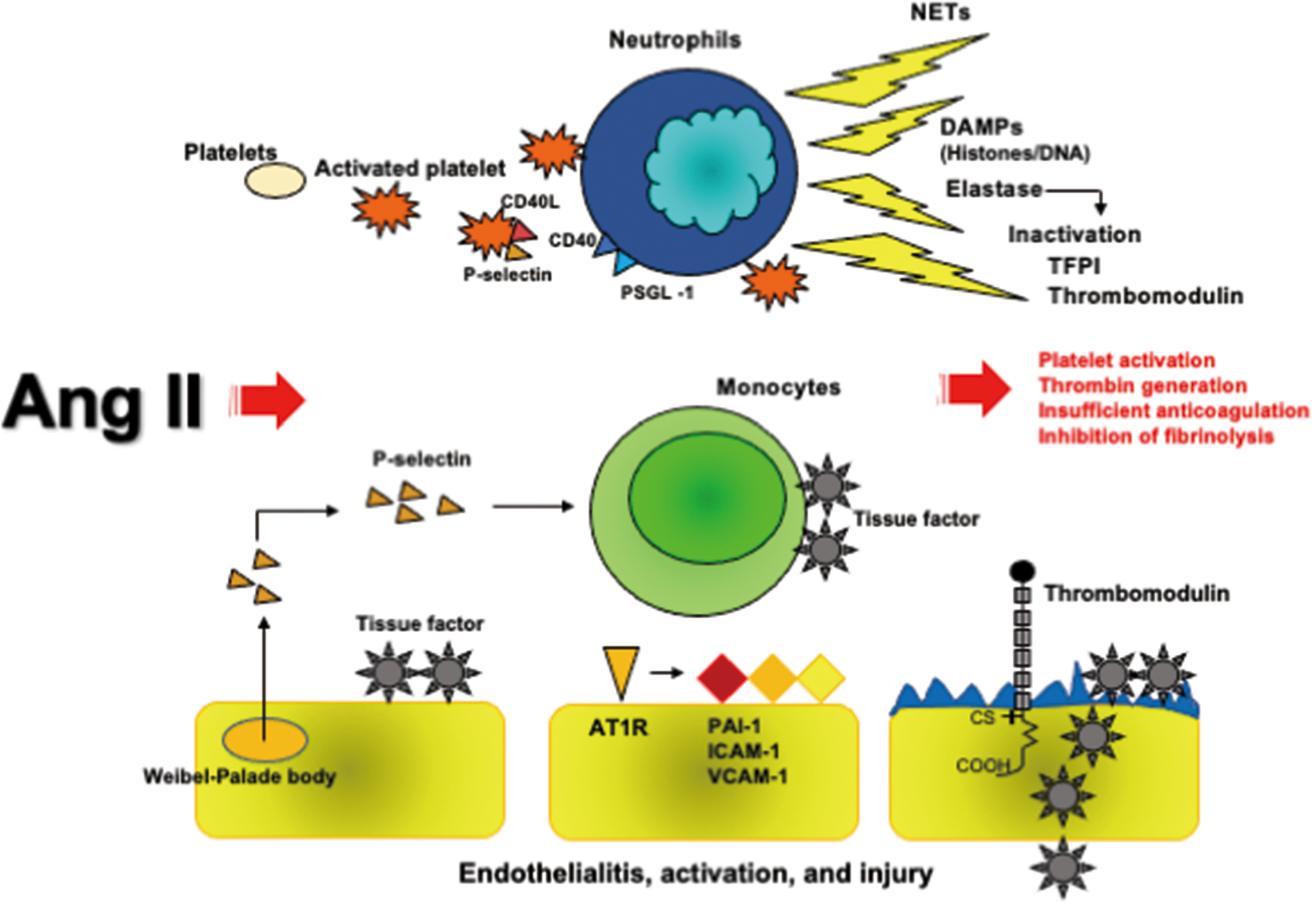
Ang II-induced coagulopathy. Ang II activates platelets expressing P-selectin and CD40L, consequently binding with CD40 and PSGL-1 expressed on neutrophils followed by NETs formation, which contain neutrophil DNA and histones decorated with elastase. Histones have a potent prothrombotic property and elastase cleaves to inactivate TFPI and thrombomodulin. Ang II and P-selectins express tissue factor on monocytes and endothelial cells. Ang II further induces the expressions of PAI-1, ICAM-1, and VCAM-1 on endothelial cells *via* AT1R. SARS-CoV-2-developed endothelialitis may reduce thrombomodulin expression like herpes simplex virus infection. All these processes caused by Ang II induce thrombus formation. Ang II, angiotensin II; AT1R, angiotensin II type 1 receptor; DAMPs, damage-associated molecular patterns; ICAM-1, intercellular adhesion molecule-1; NETs, neutrophil extracellular traps; PAI-1, plasminogen activator inhibitor; PSGL-1, P-selectin glycoprotein ligand-1; SARS-CoV-2, severe acute respiratory syndrome coronavirus 2; TFPI, tissue factor pathway inhibitor; VCAM-1, vascular cell adhesion molecule-1.

Taken together, Ang II-induced coagulopathy comprising platelet activation, thrombin generation, PAI-1 expression and endothelial injury participates in macro and microvascular thrombosis *via* AT1R in COVID-19. These processes are presented in **Figure 2**.

### Systemic Hyperfibrinolysis

#### Activation of FXIIa-Dependent Contact and Kallikrein-Kinin Systems

The bidirectional interplay between the FXII-dependent contact system and kallikrein and kinin system (KKS) is deeply involved in inflammation, coagulation, and fibrinolysis. Virus-mediated direct activation of FXII has been presumed in COVID-19, as is observed in bacterial infection (98). Hepatitis C virus RNA directly binds to FXII and FXI and augments activation of these proteases, which supports the notion that SARS-CoV-2 RNA directly activates FXII, followed by KKS activation (99). DNA purified from polymorphonuclear leukocytes assembles and activates FXII and high molecular weight kininogen (HMWK), which induces the activation of prekallikrein followed by the release of bradykinin (100). Furthermore, binding of FXII and HMWK to NET fibers comprising neutrophil DNA followed by their activation was observed in the same experiment (100). Activated neutrophil-induced NET formation has been observed in COVID-19, as mentioned above. These results, therefore, indicate that both SARS-CoV-2 RNA and NETs are likely to activate FXII-dependent contact pathways and KKS (98).

#### Inflammation and Fibrinolysis

**Figure 3:**
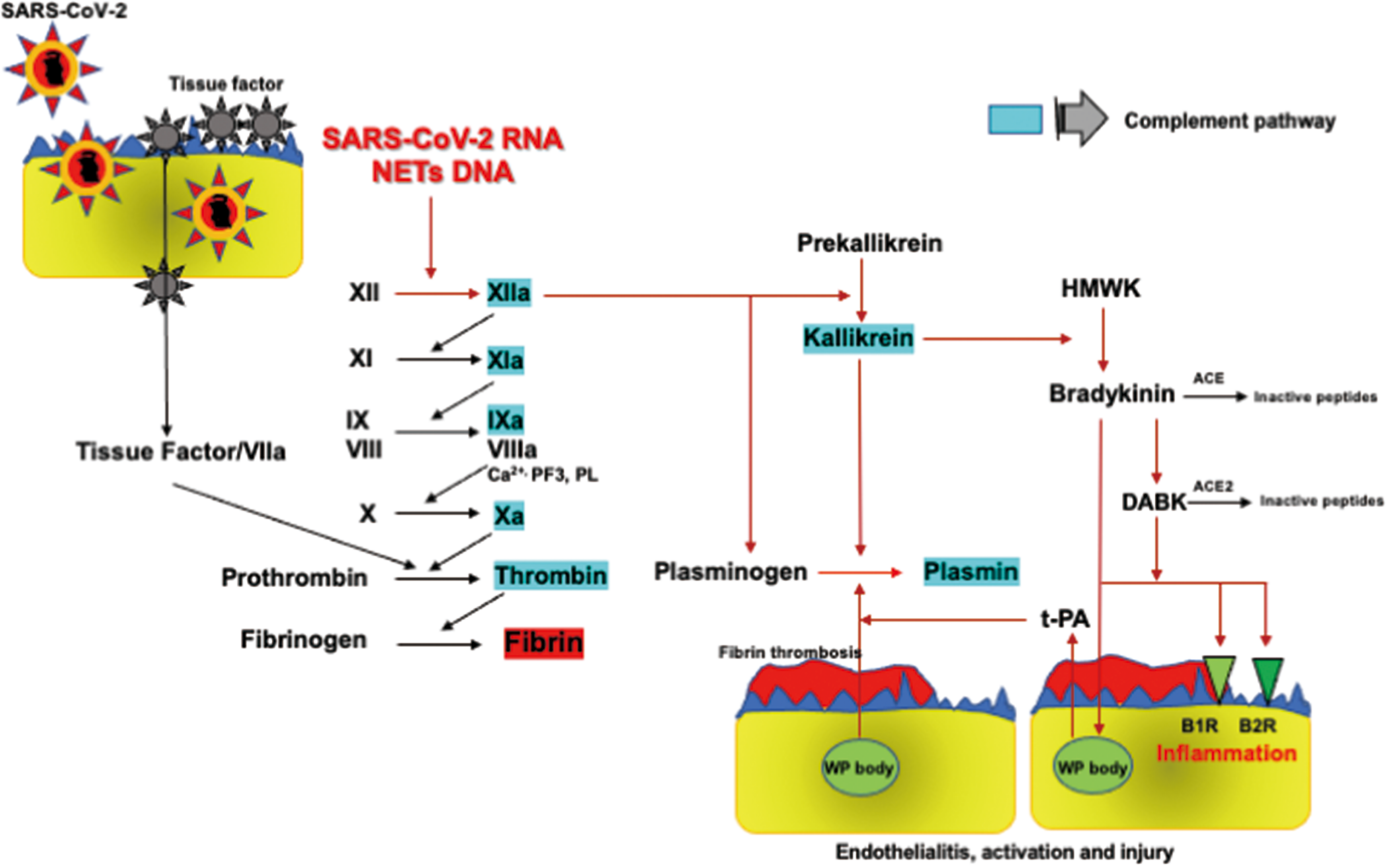
FXII-dependent contact activation system and KKS. SARS-CoV-2 RNA and NETs-containing DNA activate the FXII-dependent contact system, initiating both the contact coagulation pathway and KKS. FXIIa and kallikrein directly activate plasminogen to generate plasmin. Kallikrein cleaves HMWK to bradykinin, which is metabolized into DABK. Bradykinin and DABK induce inflammation producing inflammatory cytokines through KB1R and KB2R. Bradykinin also induces fibrinolysis through t-PA release from Weibel-Palade bodies in endothelial cells *via* KB2R, leading to plasmin formation. Therefore, bradykinin as well as FXIIa and kallikrein play central roles in inflammation and plasmin-induced fibrinolysis in COVID-19. ACE, angiotensin-converting enzyme; KB1R, kinin B1 receptor; KB2R, kinin B2 receptor; DABK, des-Arg^9^-bradykinin; HMWK, high molecular weight kininogen; KKS, kallikrein and kinin system; NETs, neutrophil extracellular traps; SARS-CoV2, severe acute respiratory syndrome coronavirus 2; t-PA, tissue-type plasminogen activator; WP body, Weibel-Palade body. Factors highlighted in blue activate complement pathways as shown in **Figure 4**.

FXIIa triggers FXI-mediated coagulation activation and also reciprocally activates prekallikrein to produce kallikrein, followed by the production of bradykinin by cleaving HMWK. ACE hydrolyzes bradykinin to inactive products whilst metabolizing it to des-Arg^9^-bradykinin (DABK), which is broken down to inactive peptides by ACE2. DABK activates the kinin B1 receptor (KB1R), whereas bradykinin binds to KB2R, both of which induce inflammation through the production of proinflammatory cytokines, TNF-α, IL-1, IL-6, and IL-8, and reactive oxygen species (19, 101). SARS-CoV-2 downregulates ACE2 with consequent increases in Ang II and causes decreases in ACE activity through a negative feedback mechanism (18, 102). Reduction of ACE activity increases in bradykinin accumulation and ACE2 downregulation impairs DBAK inactivation, both of which give rise to activation of KB1R and KB2R, followed by enhancement of inflammation (19, 101) (**Figure 3**).

The close relationships among the contact activation system, KKS, and fibrinolytic activation have long been acknowledged. FXIIa directly changes plasminogen to plasmin, and plasmin then reciprocally activates FXIIa under the condition of reduction of the α2-plasmin inhibitor (103–105). FXIIa further potentiates fibrinolytic properties through the inactivation of PAI-1 (106). FXIIa-produced kallikrein from prekallikrein also activates plasminogen into plasmin (107). Bradykinin plays a central role in the cross-talk between inflammation and fibrinolysis through the expression of inflammatory cytokines and t-PA release from endothelial cells *via* KB2R (108, 109). Influenza RNA virus induces hyperfibrinolysis, showing increased plasmin generation associated with high FDP and D-dimer levels, and low fibrinogen levels. In addition, a high FDP/D-dimer ratio of approximately 3.3 suggests fibrin(ogen)olysis due to plasmin-induced hyperfibrinolysis. Increased plasmin promotes inflammation and viral replication, which are thought to be due to fibrinolysis of immunothrombosis (110). These findings indicate that plasmin plays a pivotal role in the pathogenesis and inflammatory responses to influenza virus infection. A high FDP/D-dimer ratio of about 7.5 with low fibrinogen levels in non-survivors suggests plasmin-induced fibrin(ogen)olysis in SARS-CoV-2 infection (6). A meta-analysis of the human lung transcriptional landscape showed that ACE2 and TMPRSS2 were co-expressed with those of AT1R, kininogen, kallikrein, and fibrinogen in alveolar cells and that the t-PA gene was expressed in lung endothelial cells (111). These results support the idea of close relationships among SARS-CoV-2, ACE2, KKS, RAAS, coagulation, and fibrinolysis.

In brief, the FXII contact activation system and KKS are involved in inflammation and plasmin generation-mediated hyperfibrinolysis through insufficient ACE2, which may be an important pathomechanism in COVID-19 coagulopathy.

### DIC

The clinical characteristics of COVID-19 coagulopathy are distinct from DIC because of moderate decrease in platelet counts, mild prolongation of prothrombin time, and extremely high fibrinogen levels despite systemic thrombin and plasmin generation (50, 112, 113). Two meta-analyses, including 1,210 and 4,889 COVID-19 patients, showed that prevalence of DIC were 4.3% and 6.2%, respectively (114, 115). Pre-stage of DIC diagnosed with sepsis-induced coagulopathy score was 16.2% (114). Notably, DIC patients showed a 26.2-times higher incidence of death (log risk ratio, 3.267; confidence interval: 2.19-4.34) (115). These studies indicate the importance of high severity and late stage of the disease in developing DIC, and showed that certain patients with COVID-19 coagulopathy may proceed to fatal DIC with bleeding (6, 9, 115).

#### Cytokines

Inflammatory cytokines activate platelets and induce the expression of P-selectin, which results in tissue factor expression on monocytes and interactions with neutrophils, leading to neutrophil activation with NET release (10). Three important cytokines involved in these processes are TNF-α, IL-1, and IL-6, which were increased in COVID-19. TNF-α releases t-PA from endothelial cells followed by persistent elevation of PAI-1 playing roles in both the induction and inhibition of fibrinolysis, while IL-6 has the most relevant roles in the activation of coagulation (10, 116). IL-1 induces dysregulation of the anticoagulation pathways in addition to generating thrombin (10, 117).

#### Platelets

Platelet activation-induced P-selectin expression is a strong trigger for monocyte tissue factor expression that is a main mechanism of developing DIC (10). In severe COVID-19 patients, increased platelet activation with P-selectin expression and αIIb/β3 signaling gave rise to platelet aggregation with monocytes followed by increased tissue factor expression on monocytes (118). Moreover, D-dimer levels showed significant correlations with platelet activation for P-selectin and monocyte tissue factor expression (118), which indicate that platelet activation-induced tissue factor expression on monocytes is associated with COVID-19 coagulopathy, leading to DIC in critical cases (10, 119).

#### NLRP3 Inflammasome

Either SARS-CoV-2 itself or its RNA activate NLRP3 inflammasome *via* ACE2-Ang II-AT1R axis. This results in production of IL-1β and IL-18, which are involved in development of DIC (33–35). Platelet NLRP3 inflammasome regulates αIIb/β3 integrin outside-in signaling *via* IL-1β production, contributing to platelet activation, aggregation and thrombus formation (36). Activation of monocyte/macrophage inflammasome, and subsequent GASDMD-dependent pyroptosis-released tissue factor in the forms of microparticles, resulted in systemic blood coagulation and massive thrombosis, which were associated with high lethality in mice (37). Tissue factor-dependent coagulation activation as a main mechanism of DIC has been well acknowledged (10), therefore, inflammasome activation may be involved in the development of DIC in patients with COVID-19 (37).

#### Histones and NETs

Recent advances in the pathophysiology of DIC demonstrate the importance of pyroptosis- and necroptosis-derived histones and NETs comprising neutrophil histones, DNA, and elastase, which also act as a source of histones (120, 121). Histones and NETs trigger inflammatory cytokines release, and also initiate coagulation by tissue factor expression on monocytes and endothelial cells and by FXII activation, which is amplified by reduced anticoagulant factors and impaired fibrinolysis. Platelets are also activated by histones and NETs, leading to procoagulant phenotype *via* P-selectin expression (120, 122). Thrombin generation is extremely enhanced by prothrombinase comprising FVa and FXa, while Abrams et al. (123) found that histones act as substitutes for prothrombinase to promote FXa cleavage of prothrombin to form active thrombin and initiate DIC. *In vivo* infusion of histones causes DIC in mice associated with thrombocytopenia, consumption coagulopathy and organ dysfunction (123, 124). DIC patients, especially non-survivors, showed significantly higher histone levels than non-DIC patients, and NET formation independently predicted DIC and death in critically ill patients (123–125).

In brief, high histone levels and multiple evidence of NETs formation in various tissues and organs in COVID-19 indicate that DIC is likely to develop in critical SARS-CoV-2 infections.

#### Complement Pathways

**Figure 4:**
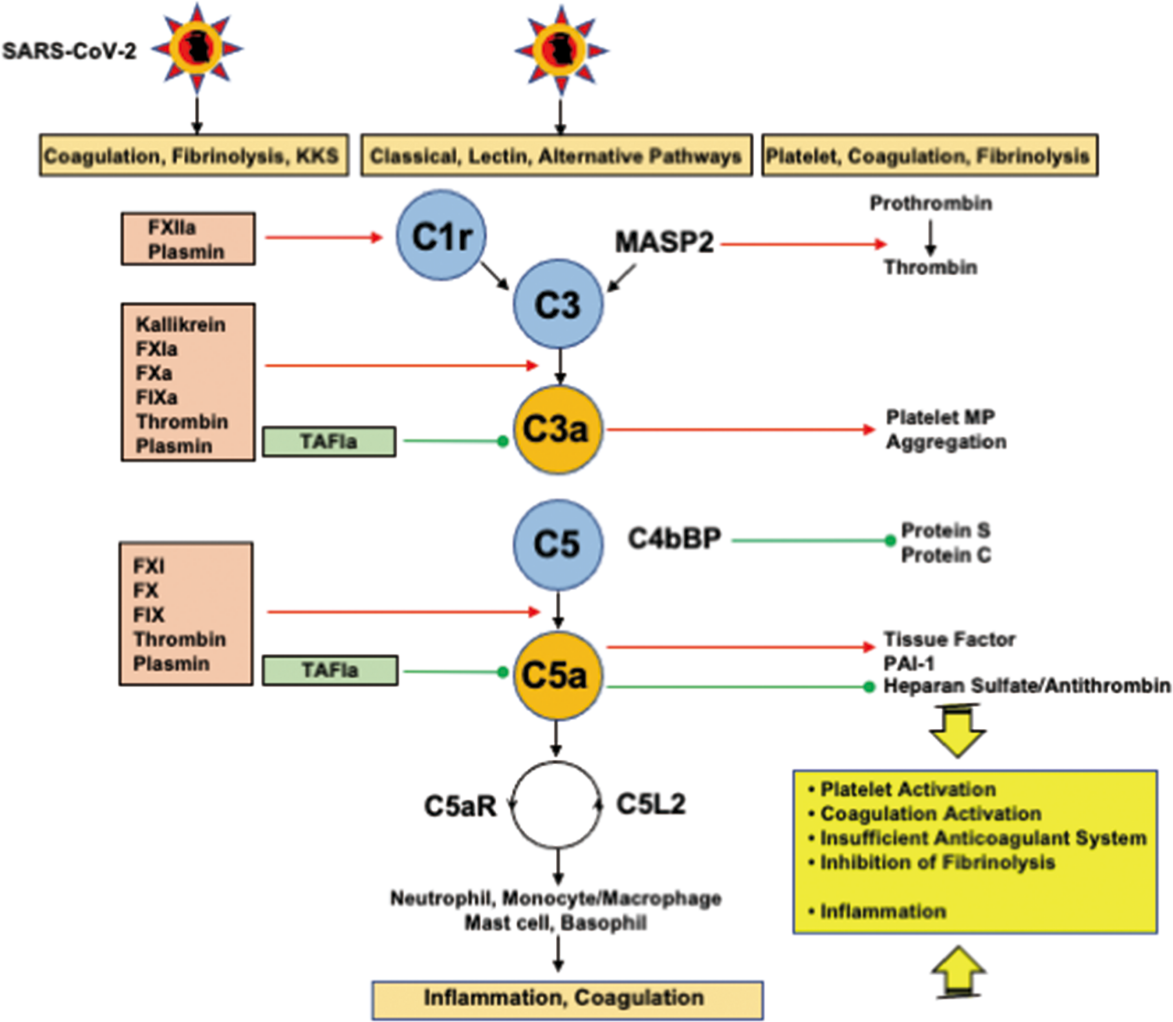
Interplays among coagulation, fibrinolysis, complement pathways, and inflammation. FXIIa and plasmin activate C1r to initiate the classical pathway. In addition to kallikrein, FXIa, FXa, FIXa, thrombin, and plasmin cleave C3 and C5 to generate C3a and C5a. Importantly, kallikrein, thrombin, and plasmin are able to directly activate C3, and thrombin can generate C5a without the participation of C3. TAFIa is the only molecule that blocks C3a and C5a. MASP2 and MAC (not shown in the figure) cleave prothrombin into thrombin. C3a generates platelet procoagulant microparticles and induce platelet aggregation. C5a expresses tissue factor and PAI-1 on neutrophils and mast cells, respectively through C5aR, and inhibits the antithrombin-mediated anticoagulation pathway through the shedding of glycocalyx heparan sulfate. Complex formation between C4bBP and protein S impairs the functional property of protein S, a cofactor of protein C, resulting in a decrease in the conversion of protein C to activated protein C. C5a finally induces inflammation *via* inflammatory cells through C5aR and C5L2. These mutual actions of the serine protease network contribute to inflammation and thrombus formation at the site of infection, if the infection is sufficiently severe, disseminating to the systemic circulation, and DIC associated with SIRS ensues. C5aR, C5a receptor; C5L2, C5-like receptor2; C4bBP, C4b binding protein; DIC, disseminated intravascular coagulation; MAC, membrane attack complex; MASP, mannose-binding lectin-associated serine protease 2; PAI-1, plasminogen activator inhibitor-1; SARS-CoV-2, severe acute respiratory syndrome coronavirus 2; SIRS, systemic inflammatory response syndrome; TAFIa, activated thrombin-activatable fibrinolysis inhibitor. Red arrows indicate activation and green lines with circle-head indicate inhibition.

The close interplays between complement pathways, coagulation and fibrinolysis systems play pivotal roles in the development of inflammation and DIC. Both C3a and C5a induce activation of coagulation, insufficient anticoagulation systems, and inhibition of fibrinolysis, which are the three major pathomechanisms of DIC (10, 126, 127) (**Figure 4**). C5a activates neutrophils, monocytes/macrophages, and endothelial cells through the C5a receptor and C5-like receptor 2, leading to inflammation, DIC, and the development of organ dysfunction (126, 127). Autopsy findings showing deposition of the membrane attack complex (C5b-9), C4d, and mannose-binding lectin (MBL)-associated serine protease 2 (MASP2) co-localized with SARS-CoV-2 spike glycoproteins in the microvasculature indicating the activation of complement pathways (128). Above studies suggest that activation of the complement pathway-generated C3a and C5a contributes to the development of DIC associated with systemic inflammation and organ dysfunction in COVID-19.

#### Phenotypes

DIC consists of thrombotic and fibrinolytic phenotypes (10, 122). DIC is a thrombotic phenotype with microvascular fibrin thrombosis associated with consumption coagulopathy (10, 129). DIC with a fibrinolytic phenotype is defined as the coexistence of DIC and systemic pathologic fibrin(gen)olysis. In other words, condition in which the same insult simultaneously causes DIC and systemic pathologic fibrin(gen)olysis is DIC with a fibrinolytic phenotype, which is usually associated with microvascular thrombosis and oozing-type bleeding (122). In COVID-19, DIC with hyperfibrinolysis due to pathologic activation of FXII and KKS belongs to the fibrinolytic phenotype.

### ARDS and the Three Viewpoints

**Figure 5:**
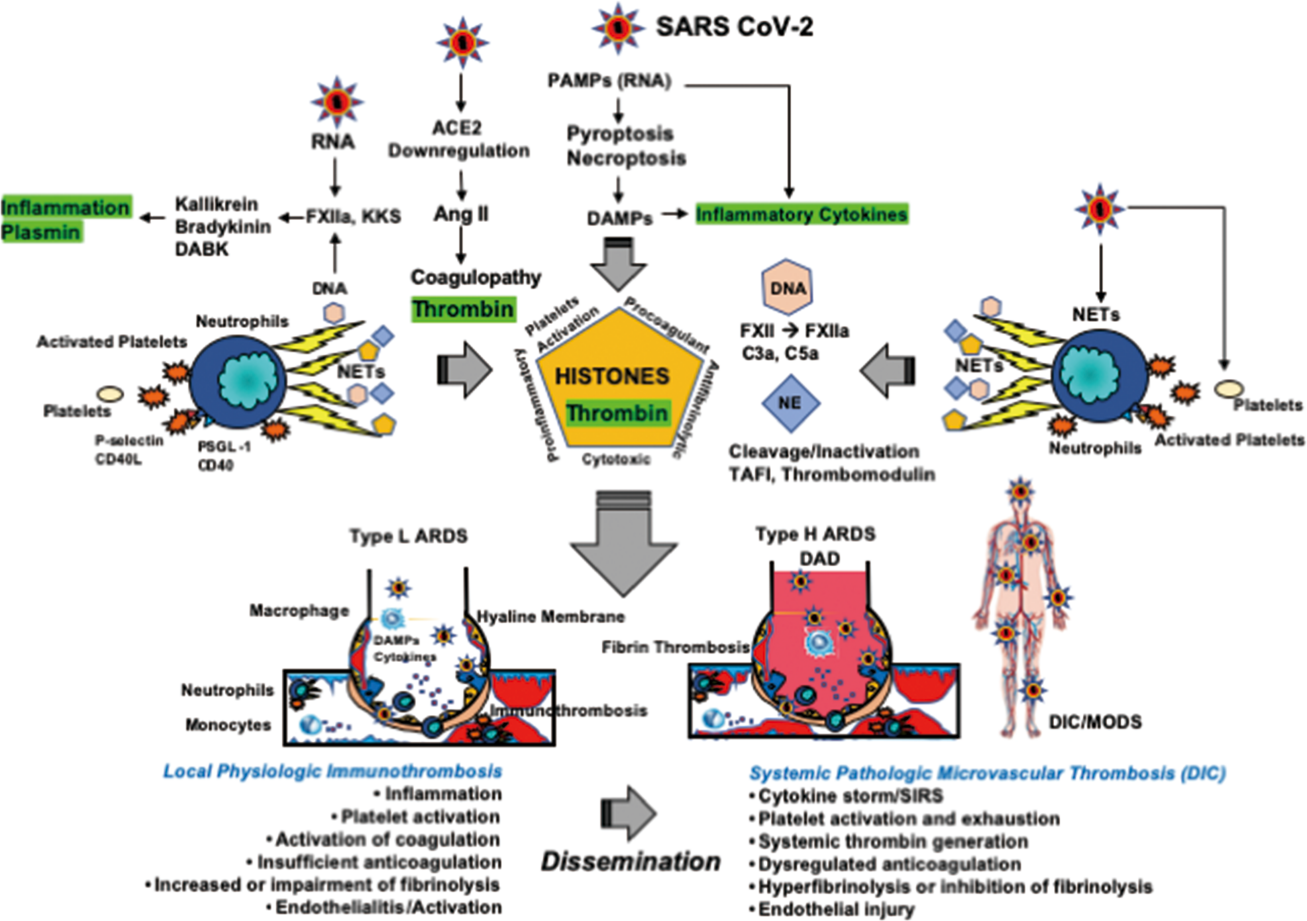
Thromboplasminflammation in COVID-19 coagulopathy and ARDS. NETs consisting of neutrophil DNA, histones, and elastase play a central role in immunothrombosis at the site of infection. DNA activates FXII to form FXIIa followed by activations of the KKS and complement pathways. Histones induce platelet activation and enhance coagulation through tissue factor expression and reduction in endogenous anticoagulants. Histones also have antifibrinolytic properties. Histones elicit inflammation by releasing TNF-α, IL-1, and IL-6 from storage pools and through NF-κB and inflammasome activations. Histones also show direct cytotoxic actions by changing intracellular Ca^2+^ load. Neutrophil elastase contributes to insufficient anticoagulation *via* the cleavage of TFPI and endothelial thrombomodulin. Therefore, the results of NETs releases are inflammation, activation of coagulation, insufficient anticoagulation, and impairment of fibrinolysis, which forms immunothrombosis at the site of infection. SARS-CoV-2 primarily targets the lungs and are entrapped by immunothrombosis, leading to type L ARDS; namely, pneumonia associated with VA/Q mismatch-induced hypoxemia with low elastance, also be called pulmonary intravascular coagulation. If the SARS-CoV-2 viral load is critical, immunothrombosis disseminates to the systemic circulation and DIC associated with SIRS ensues. At this stage, the lungs develop to type H ARDS showing diffuse alveolar damage associated with alveolar and intravascular fibrin microthrombosis, which give rise to right-to-left shunt-induced severe hypoxemia with high elastance. Mutual interactions between DIC and ARDS have been well acknowledged, which contribute to MODS. Of note, DIC is usually thrombotic due to thrombin generation and PAI-1 expression. Therefore, stimulation and inhibition of fibrinolysis are both dependent on the balance between t-PA/plasmin and PAI-1. ACE, angiotensin-converting enzyme; Ang, angiotensin; ARDS, acute respiratory distress syndrome; DABK, des-Arg^9^-bradykinin; DAD, diffuse alveolar damage; DAMPs, damage-associated molecular patterns; DIC, disseminated intravascular coagulation; KKS, kallikrein and kinin system; NE, neutrophil elastase; NETs, neutrophil extracellular traps; NF-κB, nuclear factor κB; MODS, multiple organ dysfunction syndrome; PAI-1, plasminogen activator inhibitor-1; PAMPs, pathogen-associated molecular patterns; PSGL-1, P-selectin glycoprotein ligand-1; SARS-CoV-2, severe acute respiratory syndrome coronavirus 2; SIRS, systemic inflammatory response syndrome; TAFI, thrombin-activatable fibrinolysis inhibitor; t-PA, tissue-type plasminogen activator.

SARS-CoV-2 invades lung tissue through abundantly expressed ACE2 on type II pneumocytes and endothelial cells, leading to Ang II-induced inflammation and coagulopathy. SARS-CoV-2 also gives rise to innate immune inflammation associated with leukocytes and endothelial cell activation, increasing coagulation and fibrinolysis associated with complement activation. The net results of these events include local production of inflammatory cytokines (130) and local neutrophil activation-induced immunothrombosis (56, 131) associated with pulmonary hemorrhage (68, 132). These events may explain the pathogenesis of type L ARDS (69). If SARS-CoV-2 viral loads are sufficiently high, this type of ARDS may progress to type H ARDS associated with SIRS, due to cytokine storm (42, 69, 133) and systemic microvascular thrombosis (67). A few of these cases develop DIC with a fibrinolytic phenotype characterized by increased bleeding (6, 9, 114, 115, 134). The clinical features of SARS-CoV-2 ARDS thus include increased inflammation, coagulation (involving thrombin), and fibrinolysis (involving plasmin), which can be called thromboplasminflammation. These processes are illustrated in **Figure 5**.

## Management

### Monitoring

Systemic inflammation due to proinflammatory cytokines is monitored by SIRS criteria, which discriminate 87.9% of severe sepsis patients with poor prognosis, and the number of SIRS criteria met showed proportional increases in adjusted odds of death (65). The recommended monitoring includes platelet counts, prothrombin time, fibrinogen, D-dimer, and FDP levels (7). Low platelet counts and high fibrinogen and D-dimer levels were associated with the severity of COVID-19, while prothrombin time and activated partial thromboplastin time showed no correlation with severity risk, which may be explained by increased FVIII and fibrinogen levels (8). Non-survivors in the late phase of COVID-19 showed a prominent reduction in fibrinogen associated with marked prolongation of prothrombin time, high levels of FDP, and D-dimer (6). Extremely high FDP/D-dimer ratios associated with DIC in non-survivors suggest systemic pathologic fibrin(ogen)olysis, which indicates the need for monitoring of the FDP, FDP/D-dimer ratio, and DIC score in critically ill COVID-19 coagulopathy (6). Specific and sensitive criteria should be used properly for DIC diagnosis, keeping in mind the bias in fibrinogen and prothrombin time (129, 135).

### Treatment

The first systematic review concluded that new evidence on thromboembolism in COVID-19 does not warrant a change in the present strategies on thromboprophylaxis with either unfractionated heparin or low molecular weight heparin among hospitalized patients (136, 137). For therapeutic anticoagulation, unfractionated heparin, low molecular weight heparin, or direct oral anticoagulants are recommended with a variety of regimens (136). Systematic review and meta-analyses have led to proposal of anticoagulation for prevention and therapeutic management of thrombosis in COVID-19 (138). During our analyses of published data, eight sets of guidance or guidelines have been published by a variety of societies including the World Health Organization. In addition, one multicenter international prospective registration, and four prospective randomized controlled studies have been published (139, 140). The data registry curating the largest population datasets suggests increased bleeding risk among the general population, and better results using anticoagulation therapy in those with respiratory failure requiring invasive ventilation (140). However, robust guidelines for anticoagulation in clinical practice are still needed.

From the viewpoint of three pathophysiologies of COVID-19 coagulopathy, a theoretical rationale exists in antithrombin and recombinant human thrombomodulin for COVID-19 coagulopathy. Both drugs can control inflammation. In addition, antithrombin inhibits FXIIa, FXIa, FXa, FIXa, FVIIa, thrombin, kallikrein, and C1s in the complement system (135). The target proteases of recombinant human thrombomodulin are thrombin *via* inactivation of FVa and FVIIIa. Furthermore, recombinant human thrombomodulin inhibits plasmin through activated thrombin-activatable fibrinolysis inhibitor, which further inactivates bradykinin, C3a, and C5a (10, 124, 127, 141). The binding of recombinant human thrombomodulin to histones protected platelet aggregation, fibrin thrombosis, and organ dysfunction in histone-induced DIC (124). The protease inhibitor nafamostat mesylate potently inhibits TMPRSS2, blocking spike glycoprotein-mediated fusion of the virus (142). Nafamostat mesylate is also able to inhibit other proteases such as thrombin and plasmin. Controlling plasmin is important because plasmin enhances the infectivity of SARS-CoV-2 by cleaving to the spike glycoprotein (143). All three drugs, locally approved for the treatment of DIC, may be useful for COVID-19 coagulopathy. The other promising drugs are those targeting histones and NETs, such as an anti-histone antibody, DNAse, non-anticoagulant heparin, and peptidylarginine deaminase 4 inhibitor (120). Lastly, ACE2 is a potential therapeutic target for COVID-19 coagulopathy (144).

## Thromboplasminflammation

COVID-19 coagulopathy is a disease of thromboplasminflammation consisting of Ang II-induced coagulopathy, hyperfibrinolysis by FXIIa and KKS, and DIC, all of which are associated with inflammation. Therefore, the main pathomechanisms of COVID-19 comprise overproduction of both thrombin and plasmin associated with inflammation. This could be termed thromboplasminflammation, which we define as coexistence of dysregulated thrombin generation with inflammation and systemic pathological plasmin generation in an insult. Although mechanisms are different from COVID-19 coagulopathy, coexistence of dysregulated thrombin generation and systemic pathological plasmin generation associated with fibrin(ogen)olysis has been acknowledged from the past (145).

Immunothrombosis, local thrombin generation associated with inflammation, is physiological thrombus formation at the site of infection (lungs in the case of SARS-CoV-2 infection) to kill microorganisms and inhibit their spread, in addition to impairing release of PAMPs and DAMPs, with limited host damage (70). If the infection is sufficiently severe, local immunothrombosis disseminate into systemic circulation, leading to the pathological condition called thromboinflammation, in which bidirectional interplay between thrombin and inflammation play pivotal roles (70, 122, 141). COVID-19 coagulopathy meets the definition of thromboinflammation; in addition, pathological plasmin plays important roles in COVID-19 coagulopathy. In other words, thromboinflammation and pathological plasmin generation coexist in thromboplasminflammation in COVID-19 coagulopathy.

## Open Questions and Future Perspective

A major problem of many studies on COVID-19 coagulopathy is the lack of appropriate time sequence from the onset to the death and　insufficient evaluation of disease severity using objective scores such as acute physiology and chronic health evaluation or sequential organ failure assessment. This review showed that Ang II-induced coagulopathy, hyperfibrinolysis, and DIC are three major pathomechanisms of COVID-19 coagulopathy; however, at what stage (early or late) and disease severity (mild or severe), the disease causes these three conditions – and if it is simultaneous or separate - could not be elucidated. In the late stage of severe COVID-19 coagulopathy, many confounders such as sepsis may affect the coagulopathy.

Trauma-, sepsis-, and ischemia-induced coagulopathy is an innate immune and inflammatory responses to these insults. If the insults are sufficiently severe, dysregulated inflammatory and coagulofibrinolytic response, namely DIC, ensues. The main differences in COVID-19 coagulopathy are considered to be a SARS-CoV-2-induced deterioration in the RAAS and KKS systems and direct SARS-CoV-2 infection to endothelial cells, both of which give rise to thrombosis associated with endothelialitis-induced activation and injury of the endothelium. However, clear molecular differences in the pathophysiologies of coagulopathy between viral and bacterial infections, between COVID-19 coagulopathy and other established coagulopathies of sepsis, trauma, and ischemia-reperfusion, and differences in coagulopathy within coronavirus family remain to be clarified. Although endothelialitis, endothelial activation and injury have been confirmed in COVID-19 coagulopathy, very few studies showed dynamics of anticoagulation or antifibrinolysis molecules such as antithrombin, protein C, protein S, tissue factor pathway inhibitor, and α2-plasmin inhibitor, which need to be investigated. Lastly, appropriateness of fibrinolytic and antifibrinolytic therapies for COVID-19 is still unknown. Based on the elucidation of clear molecular mechanisms of COVID-19 coagulopathy, further advances are required to establish robust treatment strategies for COVID-19 coagulopathy.

## Conclusions

The three pathomechanisms of COVID-19 coagulopathy are Ang II-induced coagulopathy, hyperfibrinolysis due to FXII and KKS activation, and DIC, which elicit thromboplasminflammation, leading to systemic inflammation, activation of coagulation and fibrinolysis associated with organ dysfunction, bleeding, and poor outcome (**Figure 5**). Controlling thrombin, plasmin, and inflammation are key to improving COVID-19 coagulopathy.

## Author Contributions

SG and TW equally contributed to this review. SG and TW planned, cowrote, improved, revised, and approved this review. All authors contributed to the article and approved the submitted version.

## Funding

This work was supported in part by JSPS KAKENHI (Grant-in-Aid (C) 2020, 20K09280).

## Conflict of Interest

The authors declare that the research was conducted in the absence of any commercial or financial relationships that could be construed as a potential conflict of interest.

## Glossary

**Table:**

ACE2	angiotensin-converting enzyme 2
ADAM17	a disintegrin and metalloprotease domain-containing protein 17
Ang I	angiotensin I
Ang II	angiotensin II
Ang(1-7)	angiotensin (1-7)
Ang(1-9)	angiotensin (1-9)
ARDS	acute respiratory distress syndrome
ASC	apoptosis-associated speck-like protein containing a caspase activating and recruitment domain
AT1R	angiotensin II type 1 receptor
AT2R	angiotensin II type 2 receptor
CD40L	CD40 ligand
COVID-19	coronavirus disease 2019
DABK	des-Arg^9^-bradykinin
DAMPs	damage-associated molecular patterns
DIC	disseminated intravascular coagulation
F	Factor (blood coagulation Factor)
FDP	fibrin/fibrinogen degradation products
GSDMD	gasdermin D
GSDMD-C	C-terminal product of GSDMD
GSDMD-N	N-terminal product of GSDMD
FXIIa	activated Factor XII
HMGB1	high mobility group box 1
HMWK	high molecular weight kininogen
ICAM-1	intercellular adhesion molecule-1
IL	interleukin
KB1R	kinin B1 receptor
KB2R	kinin B2 receptor
KKS	kallikrein and kinin system
MBL	mannose-binding lectin
MASP2	mannose-binding lectin (MBL)-associated serine protease 2
MERS-CoV	Middle East respiratory syndrome coronavirus
MLKL	mixed lineage kinase domain-like
NETS	neutrophil extracellular traps
NLRP3	NOD-like receptor family-involved pyrin domain-containing 3
NLRs	nucleotide-binding oligomerization domain-containing (NOD)-like receptors
NOD	nucleotide-binding oligomerization domain-containing
PAI-1	plasminogen activator inhibitor-1
PAMPs	pathogen-associated molecular patterns
PF4	platelet factor 4
RAAS	renin-angiotensin-aldosterone system
RIPK1	receptor interacting serine/threonine kinase 1
RIPK3	receptor interacting serine/threonine kinase 3
RLRs	retinoic acid-inducible gene I (RIG-I)-like receptors
SARS	severe acute respiratory syndrome
SARS-CoV	SARS coronavirus
SARS-CoV-2	severe acute respiratory syndrome coronavirus 2
SIRS	systemic inflammatory response syndrome
TLRs	toll-like receptors
TMPRSS2	transmembrane protease serine type-2
TNF-α	tumor necrosis factor-α
t-PA	tissue-type plasminogen activator
TRIF	toll/IL-1 receptor homology domain-containing adaptor inducing interferon-β
VCAM-1	vascular cell adhesion molecule-1
VICC	Vaccine-induced immune thrombotic thrombocytopenia
